# Early- and late anthracycline-induced cardiac dysfunction: echocardiographic characterization and response to heart failure therapy

**DOI:** 10.1186/s40959-020-00079-3

**Published:** 2020-10-13

**Authors:** Janine A. M. Kamphuis, Marijke Linschoten, Maarten J. Cramer, Pieter A. Doevendans, Folkert W. Asselbergs, Arco J. Teske

**Affiliations:** 1 Department of Cardiology, Division of Heart and Lungs, University Medical Center Utrecht, University of Utrecht, E03.511, PO Box 85500, 3508 GA Utrecht, The Netherlands; 2 Netherlands Heart Institute, Utrecht, The Netherlands; 3 Central Military Hospital, Utrecht, The Netherlands; 4 Health Data Research UK and Institute of Health Informatics, University College London, London, UK; 5 Institute of Cardiovascular Science, Faculty of Population Health Sciences, University College London, London, UK

**Keywords:** Heart failure, Anthracyclines, Cardiac dysfunction, Cardiac effects of cancer treatment

## Abstract

**Background:**

Anthracycline-induced cardiac dysfunction (ACD) is a notorious side effect of anticancer treatment. It has been described as a phenomenon of a continuous progressive decline of cardiac function, eventually leading to dilated cardiomyopathy (DCM). This progressive nature suggests that patients with a delayed ACD diagnosis have greater compromise of cardiac function and more adverse remodeling, with a poor response to heart failure (HF) treatment. This study aimed to delineate the impact of a delayed ACD diagnosis on echocardiographic characteristics and response to HF treatment.

**Methods and results:**

From the population of our cardio-oncology outpatient clinic, 92 ACD patients were included in this study (age 51.6 ± 16.2 years, median cumulative anthracycline dose 329 [200–329] mg/m^2^), and a median follow-up of 25.0 [9.6–37.2] months after ACD diagnosis. Median time to ACD diagnosis for patients diagnosed early (< 1 year) and late (> 1 year) was 4.0 vs. 47.7 months respectively. There were no echocardiographic differences between patients diagnosed early vs. late (LVEF 43.6 ± 4.9% vs. 43.0 ± 6.2% and iEDV 63.6 vs. 62.9 mL/m^2^). Eighty-three percent of patients presented with mild LV dysfunction and in 79% the LV was not dilated. Patients diagnosed early were more likely to have (partial) recovery of cardiac function upon HF treatment initiation (*p* = 0.015).

**Conclusions:**

In the setting of a cardio-oncology outpatient clinic, patients with ACD presented with a hypokinetic non-dilated cardiomyopathy, rather than typical DCM. Timing of ACD diagnosis did not impact HF disease severity. However, in patients receiving an early diagnosis, cardiac function was more likely to recover upon HF treatment.

## Introduction

Anthracyclines are potent antineoplastic drugs that constitute a cornerstone in the treatment of sarcomas, breast cancer and hematological malignancies. Shortly after the introduction of these agents in the 1960’s, cardiac dysfunction was discovered to be an important dose-limiting side effect [1]. However, despite dosage restrictions, the incidence of anthracycline-induced cardiac dysfunction (ACD) has been found to be 6% for overt heart failure and up to 18% for subclinical cardiac dysfunction [2]. The prognosis of ACD is poor, with cardiovascular mortality rates ranging from 9% at 5- and 24% at 10-years [3], up to more dramatic outcomes of 60% at 2-years in patients that have developed symptomatic HF [4].

ACD has often been classified to occur either “early” or “late”, with the first subtype developing within the 1st year after treatment and the latter more than 1 year after anthracycline-containing therapy [5]. In recent years, this subdivision has however been questioned, since a release of troponins can be detected during- or early after anthracycline administration, indicating direct damage to the myocardium upon infusion [6, 7]. The initial damage, resulting in a declined pumping capacity, will not coincide with the development of clinical manifestations of HF in a majority of patients. However, if serial echocardiographic assessment is performed, almost all asymptomatic declines are detected within the first year after anthracycline-containing chemotherapy [8]. The delayed onset of symptomatic HF may be related to the activation of compensatory mechanisms that modulate left ventricular (LV) function within a range, such that the functional capacity is preserved or only minimally depressed [9]. These compensatory mechanisms include the activation of neurohumoral systems and adaptive changes within the myocardium commonly referred to as LV remodeling. However, prolonged activation of these compensatory mechanisms ultimately has detrimental effects on the heart, leading to adverse LV remodeling with dilatation and wall thinning [10]. The onset of these changes typically marks the transition from asymptomatic- to symptomatic HF. Based on this theory, it can be postulated that over time, patients with ACD develop progressive systolic dysfunction with dilated compartments, as is the case in patients with familial DCM, long lasting left-sided valvular disease or hypertension. The aim of this consecutive cohort study was to (1) evaluate the impact of a delayed diagnosis (> 1 year after anthracycline containing treatment) on echocardiographic characteristics and (2) assess the influence of timing of diagnosis on HF treatment response.

## Methods

### Study population

In April 2015, a cardio-oncology clinic was launched at the University Medical Center Utrecht, the Netherlands, of which the protocol has been described in detail previously [11].

In short, the patient population mainly consists of patients with breast cancer or hematological malignancies who are deemed to be at increased risk for ACD due to treatment- or patient-related factors. Furthermore, cancer patients and survivors that have not received cardiac surveillance in the past (e.g. received potentially cardiotoxic treatment prior to the initiation of the clinic in 2015), are referred for screening of long-term cardiovascular complications at the discretion of the treating (hemato-)oncologist. One of the primary aims of this clinic is to facilitate a timely diagnosis of cancer therapy-related cardiac dysfunction (CTRCD) by performing serial echocardiographic assessments. When patients are diagnosed with CTRCD, guideline-based HF therapy [12] is initiated if there are signs or symptoms of HF, or when the LV ejection fraction (LVEF) reaches < 45% regardless of the presence of cardiac complaints [11].

All patients referred to the cardio-oncology clinic between April 2015 and February 2019 who were treated with anthracyclines were identified. Subsequently, we selected patients that showed impaired LV function on echocardiography or cardiac magnetic resonance (CMR). We excluded patients with LV dysfunction on multiple-gated acquisition (MUGA) scans, with echocardiographic images of insufficient quality, and patient that had received treatment with the cardiotoxic monoclonal antibody trastuzumab. Time between the initiation of anthracycline-containing therapy and ACD diagnosis was used to divide patients in two groups, namely early- (< 1 year) and late (> 1 year) ACD diagnosis, as described in the ESC Position Paper on Cancer Treatment and Cardiovascular Toxicity [5]. The study was exempted from formal approval by the Medical Ethics Committee.

### Definition of ACD

Patients were diagnosed with ACD if they met one of the following two criteria: (1) LVEF decline of ≥10 percentage points below the lower limit of normal (LLN) (< 53%) from baseline according to the European Association of Cardiovascular Imaging (EACVI) [13], or (2) an LVEF < 50% measured at > 1 time-point in case a baseline LVEF measurement prior to anthracycline treatment was not available. The diagnosis of ACD did not rely on the presence of symptoms or signs of heart failure and therefore, the selected population composes both patients with clinical and subclinical ACD.

Other possible causes of LV dysfunction were evaluated to determine the likelihood of ACD. Based on this assessment, patients were subdivided into three groups namely, ‘definite ACD’, ‘ACD with concomitant heart disease’ and ‘possible ACD’. In patients with definite ACD, alternative causes of LV dysfunction were ruled out or deemed highly unlikely. For patients that did not undergo ischemia detection, ischemic heart disease was determined unlikely in the absence of chest pain, low cardiovascular risk profile (< 2 risk factors), no coronary artery disease (CAD) on computed tomography (CT) of the chest and lack of regional wall motion abnormalities on echocardiography. Patients were diagnosed with ACD and concomitant heart disease if other causes affected LV function, but these abnormalities were determined to be insufficient to explain the degree of LV dysfunction as a whole. Lastly, in patients with ‘possible ACD’, severe valvular heart disease, left bundle branch block (LBBB), sepsis-induced cardiac dysfunction and tachycardiomyopathy were ruled out. However, in these patients, the presence of ischemic heart disease had not formally been excluded while there were signs of CAD on the chest CT. None of these patients had reported any chest complaints. The diagnosis of ACD was verified by two authors (JK and ML).

### Oncological treatment

We determined the timing of first anthracycline dose, as well as the total cumulative dose (mg/m^2^) which was calculated to an equivalent doxorubicin dose [14]. Moreover, data on mediastinal- or left-sided radiotherapy was collected.

### Echocardiographic analysis

Echocardiographs were performed by trained technicians at our cardio-oncology program. An extensive analysis was performed on the echocardiogram on which ACD had been diagnosed and on the most recent echocardiogram. All measurements were analyzed by JK, and verified by AT. Reference values and international echocardiography guidelines for echocardiographic examination can be found in Supplemental Table 1. LV volumes and -ejection fractions were preferably determined on 3D echocardiographic images. If 3D images were not available, 2D biplane (modified Simpson’s) algorithm was performed on the 4- and 2-chamber apical view. Global longitudinal strain (GLS) measurements were performed with the vendor’s software package using the 4-, 3-, and 2-chamber apical view.

### Follow-up

Patients with a follow-up of ≥6 months, or patients with complete recovery of the LVEF within 6 months, were included in the analysis of ACD reversibility. Reversibility was based on the change between the nadir- and last LVEF measurement and was classified according to the EACVI Expert Consensus [13]. In patients lacking a baseline LVEF measurements (*n* = 74), we deemed ACD to be 1) reversible in case there was an LVEF improvement of ≥10 percentage points to above the LLN, 2) partially reversible if LVEF improved 5–10 percentage points to above the LLN, and 3) irreversible if the LVEF improved < 10 percentage points from the nadir and remained below the LLN.

### Statistical analysis

Continuous data are expressed as means and standard deviations (SD) or medians and interquartile ranges [IQR]. Categorical variables are expressed as numbers (percentages). Continuous data were compared using the independent Student’s *t*-test or Mann-Whitney U. Categorical data were tested using Chi-square or Fisher exact test as appropriate. Correlation was calculated with either Pearson or Spearman correlation, where appropriate. Differences between > 2 groups were calculated using one-way analysis of variance (ANOVA) with Bonferroni post correction for multiple comparisons or the Kruskal-Wallis test. A two-sided *p*-value < 0.05 was considered significant. Statistical analyses were performed using SPSS Statistics, version 25 (IBM Corp., Armonk, NY, USA).

## Results

### Study population

**Fig. 1:**
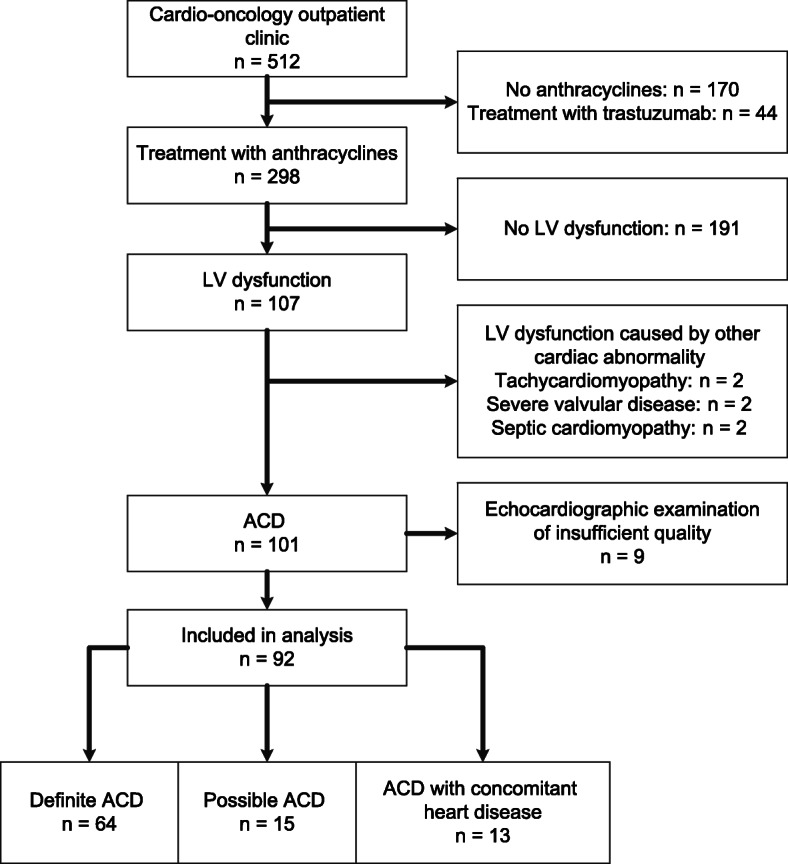
Flow chart of the study population selection

Between April 2015 to February 2019, a total of 512 patients had been evaluated at the cardio-oncology outpatient clinic (Fig. 1). Anthracyclines were administered in 342 patients, of which 44 patients were not eligible for this study due to concomitant treatment with trastuzumab. Of the remaining 298 patients, 107 (35.9%) had LV dysfunction. In six patients, the underlying cause for the impaired LV dysfunction was deemed not related to anthracyclines. Additionally, nine patients had echocardiographic images of insufficient quality to perform reliable measurements. Thereby, a total of 92 patients were included in this study. All patient characteristics at time of ACD diagnosis are outlined in Table 1. Most patients were referred for screening of cardiovascular toxicity (*n* = 78; 85%) and cardiac screening prior to stem cell transplantation was the main reason for referral (*n* = 56; 61%). Cardiac complaints, including dyspnea, angina and palpitations were the reason for referral in 14 patients (early *n* = 5; late *n* = 9).

**Table 1:** Characteristics of study participants

Demographics	Overall (***n*** = 92)	Early (***n*** = 49)	Late (***n*** = 43)	***p***-value
Male	68 (74)	38 (78)	30 (70)	
Body Mass Index (kg/m^2^)	24.5 (4.5)	24.8 (4.5)	24.1 (4.5)	0.422
Body Surface Area (m^2^)	1.9 (0.2)	1.9 (0.2)	1.9 (0.2)	0.536
Age at start cancer therapy (years)	48.2 (18.1)	52.2 (15.8)	43.7 (19.2)	0.024
Age at diagnosis ACD (years)	51.6 (16.2)	52.4 (16.1)	50.8 (16.2)	0.646
**Malignancy**				
Hematological	86 (94)	48 (98)	38 (88)	
Acute leukemia	44 (51)	32 (67)	12 (32)	
Non-Hodgkin’s lymphoma	6 (7)	1 (2)	5 (13)	
Hodgkin’s lymphoma	26 (30)	8 (17)	18 (47)	
Other^a^	10 (12)	7 (15)	3 (8)	
Breast cancer	2 (2)	0 (0)	2 (5)	
Other solid tumors^b^	4 (4)	1 (2)	3 (7)	
Cumulative anthracycline dose (mg/m^2^), [IQR]	329 [200–329]	329 [180–329]	308 [200–400]	0.114
Chest radiation	7 (8)	1 (2)	6 (14)	
**Functional class at diagnosis**				
NYHA class I + II	86 (94)	47 (96)	39 (91)	
NYHA class III + IV	6 (6)	2 (4)	4 (9)	
**Electrocardiogram**				
Heart rhythm				
Sinus rhythm	90 (98)	48 (98)	42 (98)	
Atrial fibrillation	2 (2)	1 (2)	1 (2)	
Heart axis				
Normal	82 (89)	42 (86)	40 (93)	
Left	8 (9)	5 (10)	3 (7)	
Right	2 (2)	2 (4)	0 (0)	
Ventricular conduction				
Normal	86 (93)	45 (92)	41 (95)	
Left bundle branch block	4 (4)	2 (4)	2 (5)	
Right bundle branch block	2 (2)	2 (4)	0 (0)	
**Cardiovascular risk factors**				
Hypertension	19 (21)	7 (14)	12 (28)	
Diabetes Mellitus	9 (10)	3 (6)	6 (14)	
Hyperlipidemia^c^	12 (17)	5 (13)	7 (19)	
Smoking status	*n* = 80	*n* = 42	*n* = 38	
Former smoker	31 (39)	24 (57)	7 (18)	
Current smoker	13 (16)	6 (14)	7 (18)	
Never smoked	36 (45)	12 (29)	24 (64)	
Coronary / Peripheral Artery disease	13 (14)	8 (17)	5 (12)	

Categorical variables are expressed as n (%) and continuous variables as mean (standard deviation) or median [interquartile range]; ^a^Other hematological malignancies included chronic myeloid leukemia (*n* = 4), multiple myeloma (*n* = 3), myelodysplastic syndrome (*n* = 2) and myelofibrosis (*n* = 1); ^b^Other solid tumors included sarcomas (*n* = 3) and Wilms tumor (*n* = 1); ^c^ *n* = 75 patients (38 early ACD; 37 late ACD); *ACD* Anthracycline-induced cardiac dysfunction

### Anthracycline-induced cardiac dysfunction

Sixty-four patients (70%) were diagnosed with definite ACD, and thirteen patients (14%) had ACD with concomitant heart disease(s), including LBBB (*n* = 3), CAD (*n* = 5), moderate mitral valve regurgitation (*n* = 7), non-compaction cardiomyopathy (*n* = 2) and hypertrophic cardiomyopathy (*n* = 1). In 15 patients (16%), CAD could not be excluded as additional investigations of coronary artery status had not (yet) been performed. These patients were classified as having possible ACD.

Of all 92 patients, 32 (35%) did not have any known cardiovascular risk factors, 43 (47%) had 1 risk factor, 17 (19%) had ≥2 risk factors. Forty-nine patients were diagnosed within 1 year after anthracycline-initiation (median 4 [1.9–6.4] months). ACD was diagnosed late in 43 patients (median 47.7 [41.7–87.3] months) of which 29 patients were diagnosed > 2 years after initiation of anthracycline-containing anticancer treatment. All patients had finished anthracycline-containing therapy and did not receive further treatment with cardiotoxic agents.

### Echocardiographic characterization of ACD at diagnosis

**Fig. 2:**
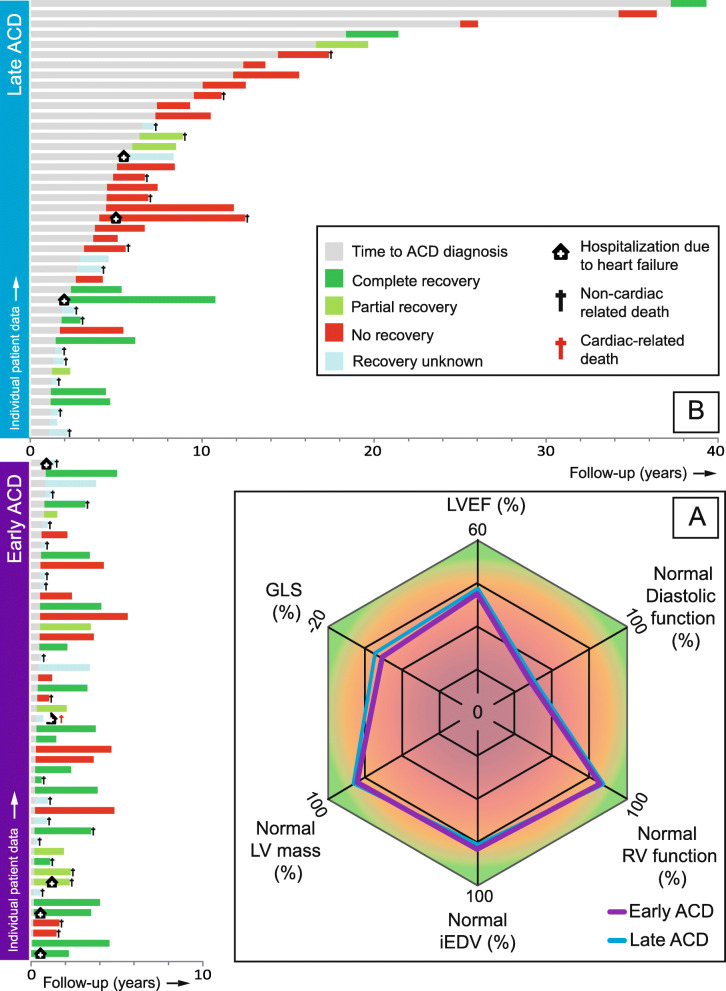
Echocardiographic characterization of early- and late diagnosed anthracycline-induced cardiac dysfunction and follow-up. **a** The radar chart shows the echocardiographic phenotype at diagnosis of early- and late ACD, which are both characterized by a mild hypokinetic, non-dilated cardiomyopathy. LVEF and GLS are expressed as group means, LV mass, iEDV and RV function are expressed as % of patients with normal outcomes and diastolic function is expressed as % of patients with diastolic dysfunction ≤ grade I; **b** Individual time periods of time to ACD diagnosis and follow-up outcomes regarding i) hospitalization due to heart failure ii) recovery of LV function and iii) (non-)cardiac death

**Fig. 3:**
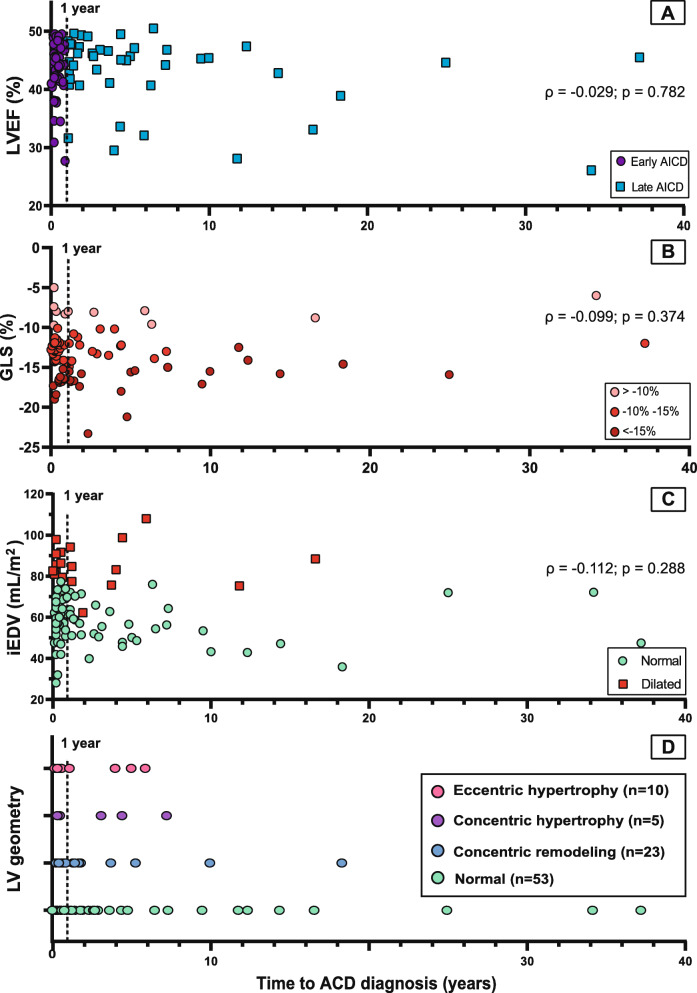
Echocardiographic outcomes at time of diagnosis. The dashed line represents the time-point of 1 year, which is used to differentiate between early- and late ACD. **a** Left ventricular ejection fraction; **b** Global longitudinal strain; **c** End-diastolic volume, indexed for body surface area and classified as ‘normal’ or ‘dilated’; **d** LV geometry, based on LV dimension and LV mass

Overall, the LVEF impairment was mild in 76 patients (83%), moderate in 12 (13%) and severe in 4 patients (4%). No echocardiographic differences were found between early- and late diagnosed ACD (Table 2, Fig. 2a). There was no relation between timing of ACD diagnosis and -LVEF (ρ = − 0.029 *p* = 0.782; Fig. 3a), −GLS (ρ = − 0.099, *p* = 0.374; Fig. 3b), or iEDV (ρ = − 0.112; *p* = 0.288; Fig. 3c) at diagnosis. Seventy-three patients (79%) had a normal indexed end-diastolic volume (iEDV) (Fig. 3c). We found a decreased, normal, and increased LV mass in 2, 74 and 15 patients, respectively. Out of the 15 patients with increased LV mass, 7 patients had hypertension and 3 patients had moderate mitral valve regurgitation. No relation between timing of ACD diagnosis and LV geometry was established (*p* = 0.710) (Fig. 3d). There was no correlation between cumulative anthracycline dose and LV mass (*r* = 0.021, *p* = 0.845). Thirty-four patients (38%) had normal diastolic function, 47 (53%) had grade I- and 8 (9%) had grade II diastolic dysfunction.

**Table 2:** Echocardiographic outcomes of patients with early- and late diagnosed ACD

	Overall (***n*** = 92)	Early (***n*** = 49)	Late (***n*** = 43)	***p***-value
**Left ventricle**				
IVSd (mm)	9.7 (1.8)	9.8 (1.7)	9.6 (1.9)	0.543
PWd (mm)	9.5 (1.6)	9.6 (1.6)	9.4 (1.7)	0.483
LVIDd (mm)	49.7 (6.8)	50.0 (7.3)	49.3 (6.3)	0.630
iLV mass (g/ m^2^)	89.9 (23.9)	91.3 (21.1)	88.4 (26.9)	0.572
Increased iLV mass (n) ^a^	15 / 91 (17)	8 (17)	7 (17)	0.319
iEDV (mL/m^2^)	63.3 (15.5)	63.6 (14.8)	62.9 (16.4)	0.840
Dilated (n) ^a^	19 (21)	9 (18)	10 (23)	0.334
iESV (mL/m^2^)	36.2 (11.2)	35.9 (9.6)	36.5 (13.0)	0.813
Dilated (n) ^a^	69 (75)	40 (82)	29 (67)	0.178
LVEF (%)	43.3 (5.5)	43.6 (4.9)	43.0 (6.2)	0.576
GLS (%)	−13.5 (3.3)	−13.2 (3.1)	−13.7 (3.5)	0.550
**Right ventricle**				
Basal RV dimension (mm)	32.8 (6.2)	32.6 (5.4)	33.0 (7.1)	0.756
RV function (*n* = 91)				
TAPSE (mm)	19.5 (4.6)	19.5 (4.3)	19.6 (4.9)	0.905
S′ (cm/sec)	12.1 (2.8)	12.2 (2.7)	11.9 (2.9)	0.632
Impaired (n) ^a^	14 (15)	7 (15)	7 (16)	0.823
Systolic RV pressure (mmHg)	21.4 (5.6)	21.0 (5.2)	21.9 (6.1)	0.617
**Diastolic function (*n*** **= 89)**				
LAVI (mL/m^2^)	28.6 (9.1)	29.1 (8.2)	28.0 (9.9)	0.598
E (cm/sec)	55.0 (18.5)	54.7 (15.80	55.5 (21.4)	0.840
A (cm/sec)	63.0 (17.0)	63.7 (17.3)	62.2 (16.9)	0.696
E/A ratio	0.91 (0.39)	0.91 (0.40)	0.91 (0.39)	0.961
E deceleration time (msec)	176 (51)	182 (60)	169 (37)	0.271
E’ (cm/sec)	8.1 (2.9)	8.0 (3.0)	8.2 (2.8)	0.747
E/E’	7.2 (2.6)	7.3 (2.5)	7.1 (2.7)	0.771

Categorical variables are expressed as n (%) and continuous variables as mean (standard deviation) or median [interquartile range]. *IVSd* end-diastolic intraventricular septal dimension, *PWd* End-diastolic posterior wall dimension, *LVEDD* Left ventricular end-diastolic dimension, *iLV* Indexed left ventricular (mass), *iEDV* Indexed end-diastolic volume, *iESV* Indexed end-systolic volume, *LVEF* Left ventricular ejection fraction, *GLS* Global longitudinal strain, *RV* Right ventricle, *TAPSE* Tricuspid annular plain systolic excursion; *S′* Doppler Tissue Imaging-derived S-wave, *LAVI* Left atrial volume index; ^a^ References values of echocardiographic measurements are provided in supplemental Table 1

### ACD treatment and clinical follow-up

The median follow-up time after diagnosis of ACD was 25.0 [10.6–38.2] months (Fig. 2b). HF medication was initiated in 68 patients (74%) (Table 3). Five of these patients were already on ACE-inhibitors or beta-blockers because of hypertension, and additional HF therapy was given. Intolerance to ACE-inhibitors and betablockers was common, and overall the maximum tolerated dose was low (Table 3). Twenty-four patients (26%) did not receive HF medication since they had an LVEF of > 45% and lacked cardiac complaints [11]. Three patients were diagnosed with ACD when they were hospitalized due to HF and 5 patients were hospitalized with decompensated HF after ACD was diagnosed. No patient was in need for cardiac mechanical support or heart transplantation during follow-up. Implantable cardioverter defibrillator (ICD) implantation was performed in 4 patients, of which 3 patients also received cardiac resynchronization therapy. One patient was successfully treated with a MitraClip because of severe, secondary mitral regurgitation, which developed after an initial decline in LV function. In total, 38 patients died during follow-up. One patient died due to acute HF. Other deaths were predominantly related to the underlying oncological disease (*n* = 30) or cancer treatment-related infections (*n* = 2). In 5 patients, the cause of death was unknown.

**Table 3:** Heart failure therapy and response to heart failure treatment

	Overall (***n*** = 92)	Early (***n*** = 49)	Late (***n*** = 43)	***p***-value
**Heart failure treatment initiation**				0.258
Initiated	63 (68)	36 (73)	27 (63)	
Adding to previous therapy	5 (5)	1 (2)	4 (9)	
LVEF > 45% and no cardiac complaints	24 (26)	12 (24)	12 (28)	
**Number of HF drugs**				0.162
1	32 (47)	20 (54)	12 (39)	
2	28 (41)	15 (41)	13 (42)	
3	8 (12)	2 (5)	6 (19)	
**ACE inhibitors / ARB**				0.538
n	61 (90)	34 (92)	27 (87)	
% of target dose	25 [16.7–50]	25 [16.7–50]	25 [25–41.5]	
**Beta-blockers**				0.183
n	39 (57)	18 (49)	21 (68)	
% of target dose	25 [12.5–50]	18.75 [12.5–25]	25 [12.5–50]	
**MRA**				
n	6 (9)	2 (5)	4 (13)	
% of target dose	37.5 [25–50]	37.5 [25–50]	37.5 [25–50]	
**Side effects**				
Hypotension	18 (26)	10 (27)	8 (26)	
Renal dysfunction	3 (4)	1 (3)	2 (6)	
Other	6 (9)	3 (8)	3 (10)	
**Duration of HF treatment (months)**	16.8 [4.8–29.8]	14.6 [3.4–28.0]	25.3 [10.1–35.2]	0.038*
**Response to HF therapy**				
	**Overall** **(*n*** **= 50)**	**(partial) recovery (*n*** **= 26)**	**No recovery (*n* = 24)**	**p-value**
**Response to HF therapy**				0.011*
Early	26 (51)	18 (69)	8 (33)	
Late	24 (49)	8 (31)	16 (67)	
**Number of HF drugs**				0.669
1	22 (44)	12 (46)	10 (42)	
2	20 (40)	9 (35)	11 (46)	
3	8 (16)	5 (19)	3 (12)	
**ACE inhibitors / ARB**				0.316
n	46 (92)	23 (88)	23 (96)	
% of target dose	25 [16.7–50]	25 [12.5–50]	25 [25–50]	
**Beta-blockers**				0.713
n	29 (59)	16 (62)	14 (58)	
% of target dose	25 [12.5–50]	25 [12.5–50]	37.5 [12.5–50]	
**Duration of HF treatment (months)**	21.9 [13.5–30.9]	22.3 [13.5–30.9]	25.9 [13.3–36.6]	0.547

Target doses are expressed as percentage of target dose according to the ESC Guideline on Heart Failure [12]. Categorical variables are expressed as n (%) and continuous variables as mean (standard deviation) or median [interquartile range]. *LVEF* Left ventricular ejection fraction, *HF* Heart failure, *ACE* Angiotensin converting enzyme, *ARB* Angiotensin II receptor blockers, *MRA* Mineralocorticoid receptor antagonist; * *p*-value < 0.05

### ACD echocardiographic follow-up and reversibility

Follow-up echocardiographic examinations were available in 67 patients, with a median follow-up time of 17.7 [10.5–26.6] months between diagnosis and the last echocardiographic examination. Of the 53 patients who had a normal iEDV at diagnosis, only 2 patients (3.7%) developed an iEDV above the upper limit of normal (ULN) during follow-up. Thirteen out of 14 patients who presented with a dilated LV showed normal iEDV values at follow-up. There was no relation between follow-up duration and the change in iEDV (*ρ* = − 0.079; *p* = 0.524).

Sixty-seven patients could be analyzed for reversibility of ACD. Of the 25 patients who were excluded from the analysis, 20 patients died before cardiac follow-up could be performed. In 32 patients (48%) no recovery of LV function was observed (treatment in *n* = 24), 10 patients (15%) had partial recovery (treatment in *n* = 9), and 25 patients (37%) showed complete recovery (treatment in *n* = 17). No differences were observed in GLS at diagnosis between patients with- and without (partial) recovery ((partial) recovery − 13.9 ± 3.0; no recovery − 14.0 ± 3.6; *p* = 0.908). Patients with early ACD were more likely to show (partial) recovery of LV function compared to late ACD patients, without significant differences in maximum tolerated doses of HF medication (Table 3).

## Discussion

The aim of this consecutive cohort study was to evaluate the echocardiographic phenotype of ACD, establish what impact a delayed diagnosis had on the degree of LV dysfunction, LV dimensions and response to HF treatment. The key findings of this study are threefold: (1) the majority of patients presented with mild LV dysfunction without LV dilatation (2) the echocardiographic phenotype was not different in patients diagnosed with early or late ACD and (3) patients with an early ACD diagnosis and prompt initiation of HF treatment were more likely to have a (partial) recovery of LV function, compared to patients with a late ACD diagnosis.

In contrast to previous studies in which ACD is described as a toxic cause of DCM [15], we found that LV dilatation was present in only one-fifth of cases at diagnosis and only two patients developed LV dilatation during a median follow-up of 17.7 months. We believe that there are three major reasons for the absence of a DCM-like phenotype. Firstly, many patients received an early diagnosis of ACD due to serial echocardiographic screening at a cardio-oncology clinic. In the past, the diagnosis of ACD was often established upon the development of symptomatic HF, and subclinical changes in LV function were not detected. In these patients, prolonged activation of compensatory mechanisms, including the renin-angiotensin aldosterone system (RAAS), and subsequent LV remodeling, may have led to a more pronounced dilatation of the LV. However, in our study, dilatation was also not present in the majority of patients with a late ACD diagnosis. The second possible reason for the absence of abnormal dimensions could be related to the reduction of cumulative anthracycline dose over the last decades. While doxorubicin doses exceeding > 500 mg/m^2^ were commonly administered in the past [1], the maximum cumulative dose of this agent is nowadays restricted to 450 mg/m^2^ [16], with a median dose of 329 [200–329] mg/m2 in this study (*n* = 9 receiving ≥450 mg/m^2^). It is plausible that these dose restrictions have resulted in an overall milder ACD phenotype. Thirdly, in patients developing LV dysfunction with an LVEF < 45%, HF treatment was promptly initiated, aiming at suppression of RAAS to prevent adverse LV remodeling. The early detection of ACD has shown to be beneficial in one study, where patients with early initiation of therapy were more likely to respond to pharmacological treatment [17]. To our knowledge, our study is the first to validate this time-dependent response to HF treatment. In conclusion, an earlier diagnosis, a restriction in the maximum cumulative anthracycline dose and the initiation of HF treatment might jointly have led to a hypokinetic non-dilated cardiomyopathy rather than DCM [18]. Based upon the results of this study, monitoring of LV function in patients at risk for ACD is recommended to detect subclinical changes in LV function as soon as possible and thereby allow for early initiation of HF treatment in case ACD develops.

We did not find any echocardiographic differences in dimension and function between patients diagnosed early vs. late (Table 2). Furthermore, there were also no discrepancies in LV mass between the two groups. To date, a number of imaging studies have evaluated this parameter in ACD both by CMR [19–21] and echocardiography [22, 23]. Currently, CMR is considered as the gold standard for measurements of cardiac structure and volumes [13]. With this technique, three studies found a decrease in LV mass [19–21], and an inverse correlation with the anthracycline dose [20]. Furthermore, a lower LV mass was predictive of cardiovascular death, appropriate ICD therapy and HF hospitalization in a multivariate model [20]. However, these findings are contradictory to echocardiographic studies which found an increase in LV mass [22, 23]. Armstrong et al. studied adults who were treated with anthracyclines during childhood. They found a reduced LV mass in nearly half of patients [24]. Comparison of CMR with echocardiography performed within 48 h, revealed that echocardiography overestimated LVEF and LV mass and underestimates LV volumes. The absence of reduced LV masses in our study population could therefore be related to LV mass measurements with echocardiography.

In contrast to our hypothesis, our data does not support the progressive nature of ACD regarding cardiac remodeling. To date, longitudinal imaging studies in patients with ACD are scarce, with a small number of studies performed in pediatric [25] and adult cancer patients [22, 26]. Lipshultz et al. prospectively followed 115 survivors of childhood acute leukemia with serial echocardiograms during a median follow-up of 11.8 years [25]. The LV contractility initially declined after doxorubicin containing chemotherapy, normalized the next 6 years and subsequently became significantly depressed > 12 years after the cancer diagnosis. In another prospective cohort study among 277 breast cancer patients treated with doxorubicin (36% in combination with trastuzumab) the LVEF decline was not progressive during a median follow-up of 2 years [22]. In a study by Jones et al. in 143 patients that were followed for 2 years, none transitioned to more advanced HF stages [26]. In our study only 5.5% of patients progressed to symptomatic HF. Nevertheless, the presence of asymptomatic LV dysfunction gives an increased risk of ultimately progressing to symptomatic HF. In a meta-analysis evaluating the risk in patients with systolic LV dysfunction due to various etiologies, the incidence of symptomatic HF was 8.4 (95% CI 4.0–12.8) per 100 person-years, compared to 1.04 (95% CI 0.0–2.2) per 100 person-years in the absence of LV dysfunction, equaling a relative risk of 4.6 (95% CI 2.2–9.8) [27]. This meta-analysis illustrates the importance of implementing effective strategies in the pre-symptomatic stages to mitigate the progression rate to symptomatic HF. This progression might also be dependent on the development of other cardiac stressors, such as hypertension, valvular disease, CAD, or the presence of pathogenic variants in cardiomyopathy-associated genes [28, 29]. In the absence of these so called “second-hits”, it is possible that a considerable proportion of patients only develops mild LV dysfunction after anthracycline-containing chemotherapy, that remains stable for years. Larger observational cohort studies, preferably with long-term follow-up can shed more light on the natural course of this specific disease entity. Also, the outcomes of the TITAN-study, which compares intensive cardiac monitoring and -treatment to usual care, will be informative on the added value of early identification of ACD [30].

### Limitations

Our analysis was restricted to patients that visited the cardio-oncology outpatient clinic. Patients treated with potentially cardiotoxic cancer therapy prior to its launch currently receive cardiological follow-up to screen for long-term cardiovascular complications. It is possible that patients who developed a more severe ACD phenotype presented at the emergency care unit earlier and were never seen in an outpatient clinic setting and therefore were not included in our study. Also, patients deemed to be at low risk for ACD do not receive cardiac screening per protocol at our cardio-oncology service. This may overall lead to an underestimation of ACD in the population of cancer patients treated with anthracyclines.

Furthermore, the current study used patient information collected during standard clinical care. Albeit the follow-up of these patients is standardized to a great extent [11], there still was heterogeneity in the data. Even though current guidelines recommend to perform cardiac screening prior to treatment [31], many patients were referred after initiation of anthracycline-treatment, and therefore lacked cardiac baseline assessment. Subtle changes in LV dimensions and –volumes within patients could therefore have been overlooked. In addition, for patients that did not undergo an echocardiography at baseline, pre-existent impaired cardiac function could have been misclassified as ACD. Also, additional testing for other causes of impaired LV function, such as CAD, had not (yet) been performed in a number of patients, leaving uncertainty of the diagnosis of ACD. Nevertheless, this represents only a small subset of our cohort where, from a clinical point of view, ACD was very likely.

Biomarkers, such as troponin or BNP, are not routinely performed at our cardio-oncology clinic and were only available in a limited number of patients. We therefore were unable to include the outcomes of biomarkers in our analysis.

## Conclusions

We found that the ACD phenotype overall was mild and a majority of patients lacked cardiac complaints. In the absence of serial echocardiographic assessment, it therefore is plausible that the impaired cardiac function would have remained undetected. Since response to HF treatment is time-dependent, detection of asymptomatic LV dysfunction is of great importance. When cardiac compromise is detected, other cardiovascular risk factors can be treated more aggressively, potentially decreasing the risk of patients progressing to more advanced HF stages. Moreover, if a patient is planned to receive further cardiotoxic treatment, preventive actions can be considered [31]. The involvement of a cardiologist in a multidisciplinary setting at cardio-oncology clinics is valuable to allow for the early detection of ACD and other adverse cardiovascular effects during cardiotoxic treatment [32].

Importantly, future research within the field of cardio-oncology should not only focus on the added value of cardiac screening, but also possible drawbacks including medicalization and increased health-care costs [33]. Trials evaluating different follow-up strategies, such as the TITAN-study [30], are required to achieve an optimal risk-benefit ratio. It is plausible that the optimal strategy varies per cancer type, as often the prognosis of the malignancy is a dominant factor. Longitudinal cohort studies establishing more insight into the natural course of ACD are herein of pivotal importance.

## Supplementary information

**Additional file 1: Supplemental Table 1.** Reference values of echocardiographic measurements and international guidelines on echocardiographic examination [16].

## Data Availability

The datasets generated and analysed during the current study are not publicly available due to protecting participant confidentiality but are available from the corresponding author on reasonable request.

## Abbreviations

ACD: Anthracycline-induced cardiac dysfunction
DCM: Dilated cardiomyopathy
LV: Left ventricle
LVEF: Left ventricular ejection fraction
iEDV: Indexed end-diastolic volume
CTRCD: Cancer therapy-related cardiac dysfunction
CMR: Cardiac Magnetic resonance
MUGA: Multiple-gated acquisition
ESC: European Society of Cardiology
LBBB: Left bundle branch block
CAD: Coronary artery disease
CT: Computed tomography
BSA: Body surface area
GLS: Global longitudinal strain
LVIDd: Left ventricular internal diameter at end-diastole
RWT: Relative wall thickness
TAPSE: Tricuspid annular plain systolic excursion
DTI: Doppler tissue imaging
EACVI: European Association of Cardiovascular Imaging
LLN: Lower limit of normal
IQR: Interquartile range
SD: Standard deviation
ANOVA: Analysis of variance
NYHA: New York Heart Association
RV: Right ventricle
ACE: Angiotensin converting enzyme
ICD: Implantable cardiac defibrillator
ULN: Upper limit of normal
RAAS: Renin-angiotensin aldosterone system
