# Supplementary material for: Early- and late anthracycline-induced cardiac dysfunction: echocardiographic characterization and response to heart failure therapy
Source: Cardiooncology. 2020 Oct 13;6:23. doi: 10.1186/s40959-020-00079-3 (PMC7557080; doi:10.1186/s40959-020-00079-3)
Supplement: Supplementary file 1 — Additional file 1: Supplemental Table 1. Reference values of echocardiographic measurements and international guidelines on echocardiographic examination [16]. [file 40959_2020_79_MOESM1_ESM.docx]

**Supplemental Table 1.** Reference values of echocardiographic measurements and international guidelines on echocardiographic examination [16].

|  | **Lower Limit of Normal** | **Upper Limit of Normal** |
| --- | --- | --- |
| **Left ventricle** | | |
| iEDV (2D)  iEDV (3D) | ♂ 34 mL/m^2^  ♀ 29 mL/m^2^ | ♂ 74 mL/m^2^  ♀ 61 mL/m^2^  ♂ 79 mL/m^2^  ♀ 71 mL/m^2^ |
| iESV (2D)  iESV (3D) | ♂ 11 mL/m^2^  ♀ 8 mL/m^2^ | ♂ 31 mL/m^2^  ♀ 24 mL/m^2^  ♂ 32 mL/m^2^  ♀ 28 mL/m^2^ |
| iLV mass* | ♂ 49 g/m^2^  ♀ 43 g/m^2^ | ♂ 115 g/m^2^  ♀ 95 g/m^2^ |
| **Left ventricular ejection fraction**  Normal  Mildly impaired  Moderately impaired  Severely impaired | ≥53%  40-52%  30-40%  <30% | |
| **LV geometry**  Normal  Concentric remodeling  Concentric hypertrophy  Eccentric hypertrophy | Normal LV mass and RWT <0.42  Normal LV mass and RWT >0.42  Increased LV mass and RWT>0.42  Increased LV mass and RWT <0.42 | |
| **Right ventricular function** | | |
| TAPSE | 17 mm |  |
| S’ | 9.5cm/sec |  |
| * Based on the Cube formula (Lang et al. 2015)  **International guidelines used for the echocardiographic analysis**   - Lang RM, Badano LP, Mor-Avi V, Afilalo J, Armstrong A, Ernande L, Flachskampf FA, Foster E, Goldstein SA, Kuznetsova T, Lancellotti P, Muraru D, Picard MH, Rietzschel ER, Rudski L, Spencer KT, Tsang W, Voigt JU. Recommendations for cardiac chamber quantification by echocardiography in adults: an update from the American Society of Echocardiography and the European Association of Cardiovascular Imaging. Eur Heart J Cardiovasc Imaging. 2015;16(3):233-70 - Nagueh SF, Smiseth OA, Appleton CP, Byrd BF, Dokainish H, Edvardsen T, Flachskampf FA, Gillebert TC, Klein AL, Lancellotti P, Marino P, Oh JK, Popescu BA, Waggoner AD. Recommendations for the Evaluation of Left Ventricular Diastolic Function by Echocardiography: An Update from the American Society of Echocardiography and the European Association of Cardiovascular Imaging. Eur Heart J Cardiovasc Imaging. 2016;17(12):1321-1360. - Lancellotti P, Tribouilloy C, Hagendorff A, Moura L, Popescu BA, Agricola E, Monin JL, Pierard LA, Badano L, Zamorano JL. European Association of Echocardiography recommendations for the assessment of valvular regurgitation. Part 1: aortic and pulmonary regurgitation (native valve disease). Eur J Echocardiogr. 2010; 11(3):223-44 - Lancellotti P, Moura L, Pierard LA, Agricola E, Popescu BA, Tribouilloy C, Hagendorff A, Monin JL, Badano L, Zamorano JL. European Association of Echocardiography recommendations for the assessment of valvular regurgitation. Part 2: mitral and tricuspid regurgitation (native valve disease). Eur J Echocardiogr. 2010;11(4):307-32 - Baumgartner H, Hung J, Bermejo J, Chambers JB, Edvardsen T, Goldstein S, Lancellotti P, LeFevre M, Miller F, Otto CM. Echocardiographic assessment of valve stenosis: EAE/ASE recommendations for clinical practice. Eur J Echocardiogr. 2009;10(1):1-25 | | |
| iEDV = indexed end-diastolic volume; iESV = indexed end-systolic volume; iLV = indexed left ventricular (mass); LV = left ventricle; RWT = relative wall thickness; TAPSE = tricuspid annular plain systolic excursion; S’ = Doppler Tissue Imaging-derived S-wave; | | |
